# Transmission of anelloviruses to HIV-1 infected children

**DOI:** 10.3389/fmicb.2022.951040

**Published:** 2022-09-16

**Authors:** Joanna Kaczorowska, Aurelija Cicilionytė, Annet Firouzi Wahdaty, Martin Deijs, Maarten F. Jebbink, Margreet Bakker, Lia van der Hoek

**Affiliations:** ^1^Laboratory of Experimental Virology, Department of Medical Microbiology and Infection Prevention, University of Amsterdam, Amsterdam, Netherlands; ^2^Amsterdam Institute for Infection and Immunity, Amsterdam, Netherlands

**Keywords:** anellome, anelloviridae, anellovirus, early-life infections, mother-to-child transmission, virome

## Abstract

Anelloviruses (AVs) are widespread in the population and infect humans at the early stage of life. The mode of transmission of AVs is still unknown, however, mother-to-child transmission, e.g., via breastfeeding, is one of the likely infection routes. To determine whether the mother-to-child transmission of AVs may still occur despite the absence of natural birth and breastfeeding, 29 serum samples from five HIV-1-positive mother and child pairs were Illumina-sequenced. The Illumina reads were mapped to an AV lineage database “Anellometrix” containing 502 distinct ORF1 sequences. Although the majority of lineages from the mother were not shared with the child, the mother and child anellomes did display a significant similarity. These findings suggest that AVs may be transmitted from mothers to their children via different routes than delivery or breastfeeding.

## Introduction

Anelloviruses (AVs) are small, circular single-stranded DNA viruses. So far, AV infection has not been associated with any disease, however, the levels of AVs seem to be connected with the levels of host immunosuppression (Focosi et al., 2016). AVs are detected in a majority of body fluids in people of all ages (Spandole et al., 2015), but they dominate in blood, being one of the most abundant viruses in this environment (Cebriá-Mendoza et al., 2021). Not much is known about the ways of transmission of AVs. There is evidence of blood transfusion transmission (Arze et al., 2021). However, considering the enormous abundance of AVs in the human population, including young children, a mother-to-child transmission is highly likely.

Children are born AV-negative (Lim et al., 2015; Liang and Bushman, 2021), and there was so far no convincing evidence of vertical transmission of AVs (Tyschik et al., 2017). AV DNA was detected in children’s blood (Tyschik et al., 2017, 2018; Kaczorowska et al., 2022a) and feces (Lim et al., 2015) in the first months of life, thus it is likely that AVs are transmitted from the mother to child during the delivery or the post-partum period. Children born naturally tend to have higher loads of AVs compared to the ones born via the caesarian section (McCann et al., 2018). Moreover, AV DNA has been detected in breastmilk (Ohto et al., 2002; Maqsood et al., 2021), and we described recently that beta- and gammatorqueviruses are dominating both in breastmilk and in the blood of children younger than 6 months, which suggests a role of breastfeeding in AV transmission (Kaczorowska et al., 2022a). However, other means of mother-to-child transmission, such as fecal-oral or respiratory, are yet to be explored.

Here, we Illumina-sequenced a total of 29 serum samples derived from five mother and child pairs. We compared the viromes of the mother and child pairs and assessed the presence of shared anellovirus genomes. All the subjects were HIV-1 positive, thus the children were born via cesarean section and they were not breastfed.

## Materials and methods

### Clinical samples

Twenty-nine serum samples were collected from five mothers and their children. All individuals were HIV-1 positive. There was no information available regarding the presence of antiretroviral therapy in the subjects at the moment of samples’ collection, except the mother 3, who was on the therapy from the second time point onward. Four mothers (M1, M3, M4, and M5) and two children (C1 and C5) had more than one serum sample available, and the samples were collected longitudinally at irregular intervals. All samples are listed in Supplementary Table 1.

### Nucleic acid isolations

After thawing at room temperature, the serum samples were centrifuged for 10 min at 5,000 *g*. A 100 μL of supernatant was transferred to a new tube and treated with TURBO DNase (Invitrogen). The total nucleic acids were extracted using the Boom isolation method (Boom et al., 1990), and stored at –80°C until further use.

### Genus-specific qPCR

Three quantitative PCRs (qPCRs) were performed on all the selected samples to assess the prevalence and concentration of AV genera in the tested samples. The first qPCR detected the genus alphatorquevirus, the second one detected betatorquevirus, and the third—beta- and gammatorquevirus. The qPCRs were performed as described previously (Kaczorowska et al., 2022a). Briefly, the qPCR reaction mixture consisted of 2.5 μL isolated nucleic acids (non-rolling circle amplified), 6.25 μL 2× Qiagen RotorGene Probe Master-mix (Qiagen, catalog number 204574), 0.25 μL probe, 0.5 μL forward, and 0.5 μL reverse primer (all 10 μM) and 2.5 μL H_2_O. The reaction was performed on a Rotor-Gene machine (Qiagen GmbH, Hilden, Germany) as follows: 95°C for 3 min, followed by 40 cycles of 95°C for 3 s, 60°C for 10 s. The final elongation step was held at 72°C for 3 min.

### Illumina libraries preparation

The Illumina libraries were prepared as described previously (Kaczorowska et al., 2022b). Briefly, the nucleic acid samples were first amplified using rolling circle amplification (4 h at 30°C, followed by 10 min at 65°C) and then fragmented using fragmentase enzyme, for 25 min at 37°C. The ends of the fragmented nucleic acids were repaired using polymerase I, large (Klenow) fragment (New England Biolabs, NEB) in combination with NEB2 10× buffer (NEB) and dNTPs (final concentration 500 μM each). A-taling was performed with 3′-5′ exo (-) polymerase I, large (Klenow) fragment (NEB), NEB2 buffer, and dATPs (final concentration 200 μM, NEB). Both reactions were performed at 37°C for 30 min and between each of the mentioned enzymatic reactions, an AMPure XP Beads clean-up was performed. The NEB Next adaptors were mixed with T4 ligase (Invitrogen) and T4 buffer and the reaction was incubated overnight at 16°C. Afterward, an AMPure XP Beads size selection was performed and the eluate was used in the adaptor-enrichment PCR. The PCR master mix consisted of Q5 Hot Start master mix (NEB), NEB Next universal primer (final concentration of 0.5 μM; NEB), NEB Next index primer (unique for each sample; final concentration of 0.5 μM; NEB), and USER enzyme (NEB). Cycling was performed as follows: 37°C for 15 min, 98°C for 30 s, followed by 12 cycles of 98°C for 10 s and 65°C for 75 s, followed by a final extension at 65°C for 5 min. Two rounds of AMPure XP Beads size selection were performed, and the concentration of each sample was measured using Qubit High Sensitivity assay (Invitrogen). The samples were pooled at equal concentrations and run on the Illumina miSeq sequencing machine. The pool containing samples from mothers (*n* = 22) was processed and ran separately from the children’s sample pool (*n* = 7), to avoid cross-contamination.

### Anellometrix sequence database

All complete or nearly complete human-derived AV sequences were downloaded from NCBI database (state for October 2021). The sequences were merged into one fasta file, and the *ORF1* gene nucleotide sequences were extracted using EMBOSS getorf.^1^ The sequences were clustered using 95% threshold, and any duplicates were removed using dedupe script from BBTools.^2^ By running a BLASTn search against a recently updated database of AV sequences (Varsani et al., 2021), we categorized the majority of the lineages into species. The final Anellometrix genome database consisted of 502 *ORF1* sequences (Supplementary Table 2).

### Sequencing data processing

The adaptors and low quality reads were trimmed using Trimmomatic version 0.39 (Bolger et al., 2014). Paired reads were aligned to the Anellometrix database using bowtie2 (Langmead et al., 2009), using –very-sensitive setting. The obtained sam files were indexed, converted to bam, and sorted using SAMtools (Li et al., 2009). A table with mapped read numbers was generated using SAMtools idxstats function, and the percentage coverage was obtained using bbmap mpileup command.^3^ The mapping was considered valid when the genome was covered at least once for ≥75% of the genome length. The read counts were normalized to reads per million (RPM), or, for the heatmap graphs, to relative abundance.

To compare the anellomes between the samples, we used unweighted UniFrac on QIIME2 (Lozupone et al., 2006; Bolyen et al., 2019). Unweighted UniFrac calculates the distance matrix based on presence-absence tables and phylogenetic relationships between the tested samples. The ORF1 sequences of Anellometrix database were aligned using MAFFT (E-INS algorithm) (Katoh et al., 2018) and the maximum-likelihood phylogenetic tree was constructed using RAXML (Stamatakis, 2014). All the abovementioned programs were run on a high-performance cluster computer Lisa (Surfsara).^4^

### Statistical analysis

All graphs were constructed in R version 4.1.3 using the tidyverse, vegan, reshape2, and ggpubr packages, and the statistics were calculated using rstatix and vegan packages.

## Results

**FIGURE 1:**
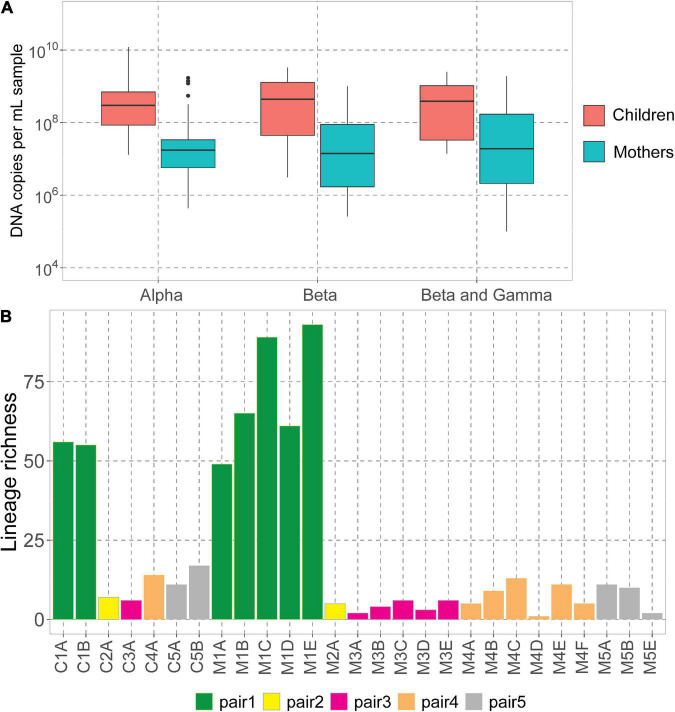
Anelloviruses in mothers and their children. **(A)** Alpha-, beta-, and beta- and gammatorquevirus DNA copies per mL serum in mothers and children measured by genus-specific qPCR; only samples positive in the assay are included in the graph. Wilcoxon sum-rank test was used to assess the significance; all differences were insignificant (*p*-value >0.05). **(B)** Number of lineages (richness) in each sample.

We assessed the DNA concentrations of the 3 human-infecting anellovirus (AV) genera using the genus-specific qPCRs. All samples were positive in alphatroquevirus qPCR, while five samples were negative in betatorquevirus and four in beta- and gammatorquevirus qPCR (Supplementary Table 1). Interestingly, we observed negative values only in mother samples after having given birth, but there were no significant differences between AV DNA concentrations between mother and child samples (Figure 1A).

The highest AV DNA concentrations among mothers were noted for mother #1 (Supplementary Figure 1). Alphatorquevirus DNA loads were stable for all mothers (Supplementary Figure 1A), while beta- and gammatorquevirus loads were fluctuating over time (Supplementary Figures 1B,C).

Illumina sequencing resulted in a total of 5.4 × 10^7^ paired reads, ranging from 3.0 × 10^5^ (sample M1A) and 6.5 × 10^6^ (C3A) reads per sample (Supplementary Table 1). Sample M5D was excluded from further analysis due to a low number of reads. We mapped the reads to the Anellometrix database, which consists of 502 ORF1 sequences derived from a broad spectrum of AV lineages. We obtained between 0 reads per million (rpm; sample M5C) and almost 5 × 10^5^ rpm (C1B) reads mapping to Anellometrix reference sequences. The AV richness differed among the subjects, with the highest richness observed in mother #1 samples (Figure 1B), which also showed the highest AV DNA concentrations in genus-specific qPCRs (Supplementary Figure 1). One of the samples from this subject, M1E, had reads mapping to as many as 98 distinguishable AV lineages (Figure 1B). Interestingly, of all children, child #1 also possessed the highest number of lineages (56 lineages). The lowest richness values were observed in child #2 (7 lineages)/mother #2 (5 lineages), and child #3 (6 lineages)/mother #3 (6 lineages). One of the samples derived from mother #5 (M5C) had a proper number of sequence reads, a reasonably high virus load (10^7^ DNA copies/mL), yet no detectable AV Illumina reads. Apparently, the library preparation did not work in this sample, possibly due to a lack of circular genomes needed for rolling circle amplification. There was no significant difference between the number of lineages detected in mothers and in children (Wilcoxon sum-rank test, *p*-value >0.05).

**FIGURE 2:**
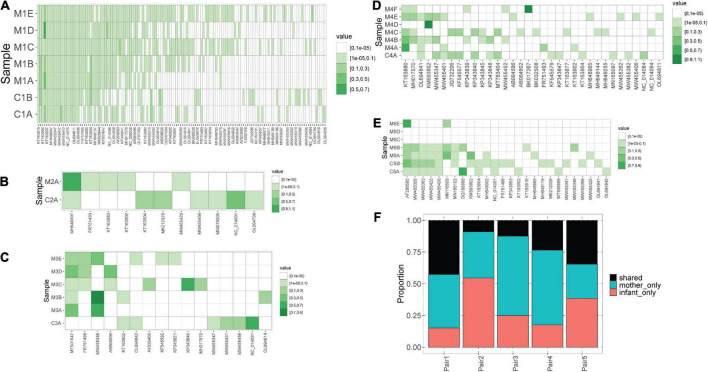
Blood anellomes of mothers and children. Heatmaps of relative abundance of different anellovirus lineages (*x*-axis) in samples (*y*-axis) within pair #1 **(A)**, pair #2 **(B)**, pair #3 **(C)**, pair #4 **(D)** and pair #5 **(E)**. The stronger the color, the higher the relative abundance (indicated by “value”), and the white color indicates the absence of a lineage. **(F)** Proportion of shared and not shared lineages within each pair. The unshared lineages were either detected solely in the mother (“mother-only”) or in the child (“infant-only”).

We observed that mothers and children within the same pair possessed a similar number of lineages (Figure 1B), however, the lineages were not always the same (Figure 2). In pair #1, which had the broadest anellome, 59 out of the total of 138 (43%) lineages were shared between mother and children samples (Figure 2A). There was just 1 lineage shared between the mother and the child in pair #2 (out of 11 lineages; 9%; Figure 2B), 2 out of 16 in pair #3 (12.5%; Figure 2C), 8 out of 34 in pair #4 (24%; Figure 2D), and 9 out of 26 in pair #5 (35%; Figure 2E). For pairs #1, #3, and #4 the majority of not shared lineages were present in mothers only, while in pairs #2 and #5 most of the unshared lineages were detected in infants (Figure 2F). In pair #1 and pair #5, the shared lineages were significantly more abundant in mother samples than the unshared lineages (Wilcoxon sum-rank test; *p*-value = 7.3 × 10^–11^ for pair #1 and 0.01 for pair #5; Supplementary Table 3). Moreover, the most abundant AV lineage of mother #2 was also the only one that was shared with the child (Figure 2B). No correlation between the relative abundance and sharing of the lineage was observed in pairs #3, #4, and #5 (*p*-value >0.05).

Of note, we observed that anellomes of the mothers that had more than 1 sample available (#1, #3, #4, and #5) were relatively stable in time in terms of the anellome breadth. The broad anellome of mother #1 remained so for the whole follow-up period (Figure 2A), while the smaller anellomes of mothers #3 (Figure 2C) and #4 (Figure 2D) were narrow throughout the follow-up. However, only a small number of lineages persisted throughout the whole follow-up period. In mother #1, 37 out of 117 lineages were detected in all the time points, in mother #3 it was 1 out of 16, and there were no such lineages in mother #4 and mother #5. Moreover, in mother #5, a large drop in the number of lineages was observed over time—with samples M5C and M5D having no detectable lineages, and the last sample possessing only two lineages (Figure 2E).

**FIGURE 3:**
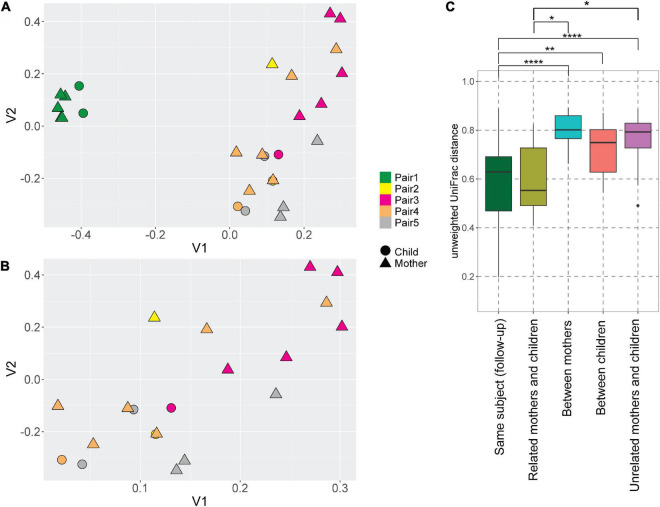
UniFrac distances of the anellomes. Principal Component Analysis (PCoA) plot using unweighted UniFrac distances between all samples **(A)** and samples excluding pair #1 **(B)**. The colors represent different pairs, while the shapes represent the category of a sample (child or mother). **(C)** Unweighted UniFrac pairwise comparison between the longitudinal samples from the same subject, and related and unrelated samples (thus within or outside the pairs). To compare related and unrelated samples, only selected samples (Supplementary Table 1—samples marked with an asterisk) were used. Statistical significance was assessed using Wilcoxon sum-rank test. Only the significant values are marked on the graph; explanations of the symbols: *P ≤ 0.05, **P ≤ 0.01, ****P ≤ 0.0001.

Even though the majority of AV lineages were not shared between the mothers and children, we still hypothesized that the AV lineages of mother and child within a pair are phylogenetically related, and unrelated samples possess more phylogenetically divergent anellomes. We compared the anellome diversity and the phylogenetic relationships of all samples using the unweighted UniFrac. Principle component analysis (PCoA) of pairwise unweighted UniFrac showed strong clustering of samples from pair #1 (Figure 3A), but no such clustering was observed in the case of samples from the remaining pairs (Figure 3B).

Next, we compared the unweighted UniFrac distances between the samples (Figure 3C). Unsurprisingly, the mean pairwise distances between the longitudinal samples derived from the same subject were significantly smaller than the ones measured between different subjects: different mothers (Wilcoxon sum-rank test; *p*-value = 9.34 × 10^–8^), and different children (*p*-value = 0.01; Figure 3C, Supplementary Table 4). Thus, to avoid the bias caused by the high similarity of follow-up samples from the same subject, we selected just one sample per subject when comparing the related and unrelated mothers and children. We chose the mother and child samples within each pair that were closest to each other in terms of UniFrac distances (samples marked with asterisks in Supplementary Table 1). We selected these samples because they were most likely collected at the moment of the most prominent mother-to-child transmission. There was indeed a significant difference in pairwise distances between mothers and related children versus unrelated mothers and children (*p*-value = 0.04; Figure 3C; Supplementary Table 4). This result shows that even though children do not share exactly the same AV lineages with their mother, their anellome composition is more similar to their own mother compared to unrelated mothers or children. Next to that, we also compared the UniFrac distances using mother samples collected closest to the delivery moment (samples marked by “$” in the Supplementary Table 1). In this case, we lost significance between related samples on the one hand and unrelated samples on the other hand (*p*-value >0.05; Supplementary Figure 2, Supplementary Table 5). This finding suggests that the moment of delivery is not the main moment of AV transmission.

## Discussion

In this study we compared the anellovirus (AV) viromes (anellomes) of 5 HIV-1 infected mother and child pairs. We observed that related mothers and children possess more similar anellomes than unrelated people—both in the terms of AV lineage richness and phylogenetic relationships. We found an inconsistent number of shared lineages within pairs, which suggests that there may not be a general pattern of transmission when children are born from HIV-1 infected mothers. One mother and child pair shared almost half of the lineages (pair #1), while another shared as little as 1 out of 11 lineages (pair #2), and another only 2 out of 16 (pair #3). We hypothesize that the AV lineages that were present only in children may have been acquired from siblings, other family members tending to the child, or from the environment.

The healthy human virome is regarded as highly dynamic in the first months of life, both in the gut and in blood (Lim et al., 2015; Tyschik et al., 2017; Bushman and Liang, 2021; Kaczorowska et al., 2022a). The AVs colonize infants within the first 6 months of life (Lim et al., 2015; Kaczorowska et al., 2022a), yet the main source or route of the first infection is still unknown. All children in our study were born via caesarian section, were not breastfed, and still were infected by AVs in the very first months of life—thus the transmission must have occurred via a different route. Fecal-oral transmission is likely—feces are positive for AVs both in children and in adults (Lim et al., 2015; Shkoporov et al., 2019; Liang and Bushman, 2021; Beller et al., 2022). Airway transmission is another possible route since AVs are frequently detected in nasal secretions (Maggi et al., 2003), saliva (Inami et al., 2000; Naganuma et al., 2008; Liang and Bushman, 2021), and bronchoalveolar lavage liquid (Young et al., 2015; Segura-Wang et al., 2018). In one longitudinal study by Maggi and colleagues, two children were initially PCR-positive for alphatorquevirus DNA in the nasal secretions and negative in plasma. After a month since the initial detection in the respiratory tract, both children became positive for alphatorquevirus in plasma (Maggi et al., 2003), which suggests that the airway may be an important transmission route of AV infection in children. A metagenomic study involving paired nasal, fecal, and blood samples from mothers and their children would shed more light on the importance of the airway and fecal-oral transmission routes in early-life AV acquisition.

## Data availability statement

The datasets presented in this study can be found in online repositories. The names of the repository/repositories and accession number(s) can be found below: https://www.ncbi.nlm.nih.gov/, PRJNA838480.

## Ethics statement

The studies involving human participants were reviewed and approved by Medical Ethics Committee of the Amsterdam University Medical Center (location AMC) of the University of Amsterdam, Netherlands (MEC 07/182). Written informed consent to participate in this study was provided by the participants’ legal guardian/next of kin.

## Author contributions

JK, AC, AW, and LH designed the study. MB selected and provided the samples and extracted the available metadata. JK, AC, AW, MD, MFJ, and MB performed the wet lab experiments. AC, AW, and JK performed the analysis of next-generation sequencing results, designed the graphs, and performed the statistical analysis. JK wrote the initial draft. LH reviewed and edited the manuscript and supervised the experiments. All authors contributed to the manuscript revision.

## Footnotes

1
http://emboss.bioinformatics.nl/cgi-bin/emboss/getorf

2
https://jgi.doe.gov/data-and-tools/software-tools/bbtools/

3
https://sourceforge.net/projects/bbmap/

4
https://www.surf.nl/
