# Supplementary material for: Transmission of anelloviruses to HIV-1 infected children
Source: Front Microbiol. 2022 Sep 16;13:951040. doi: 10.3389/fmicb.2022.951040 (PMC9523257; doi:10.3389/fmicb.2022.951040)
Supplement: Supplementary file 2 [file Data_Sheet_1.docx]

Supplementary Material

# Supplementary Figures


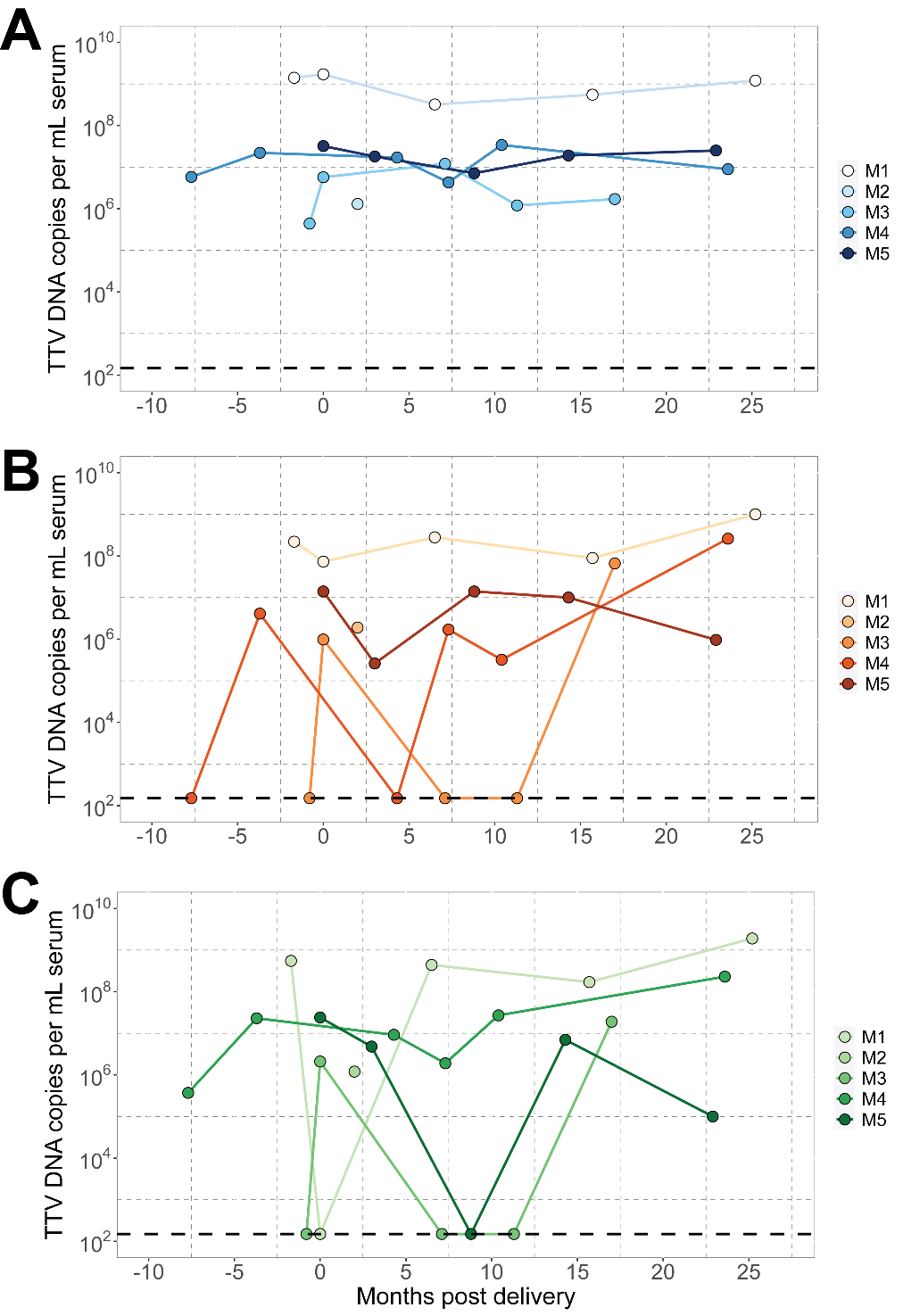


**Supplementary Figure S1.** Anellovirus load over time in mothers. Results of genus specific qPCRs in time for the mother samples: A) qPCR detecting alphatorquevirus, B) betatorquevirus, C) beta- and gammatorquevirus. The minus sign next to the value of the x axis indicates the months before the delivery. The dashed line indicates the detection limit of the assay.


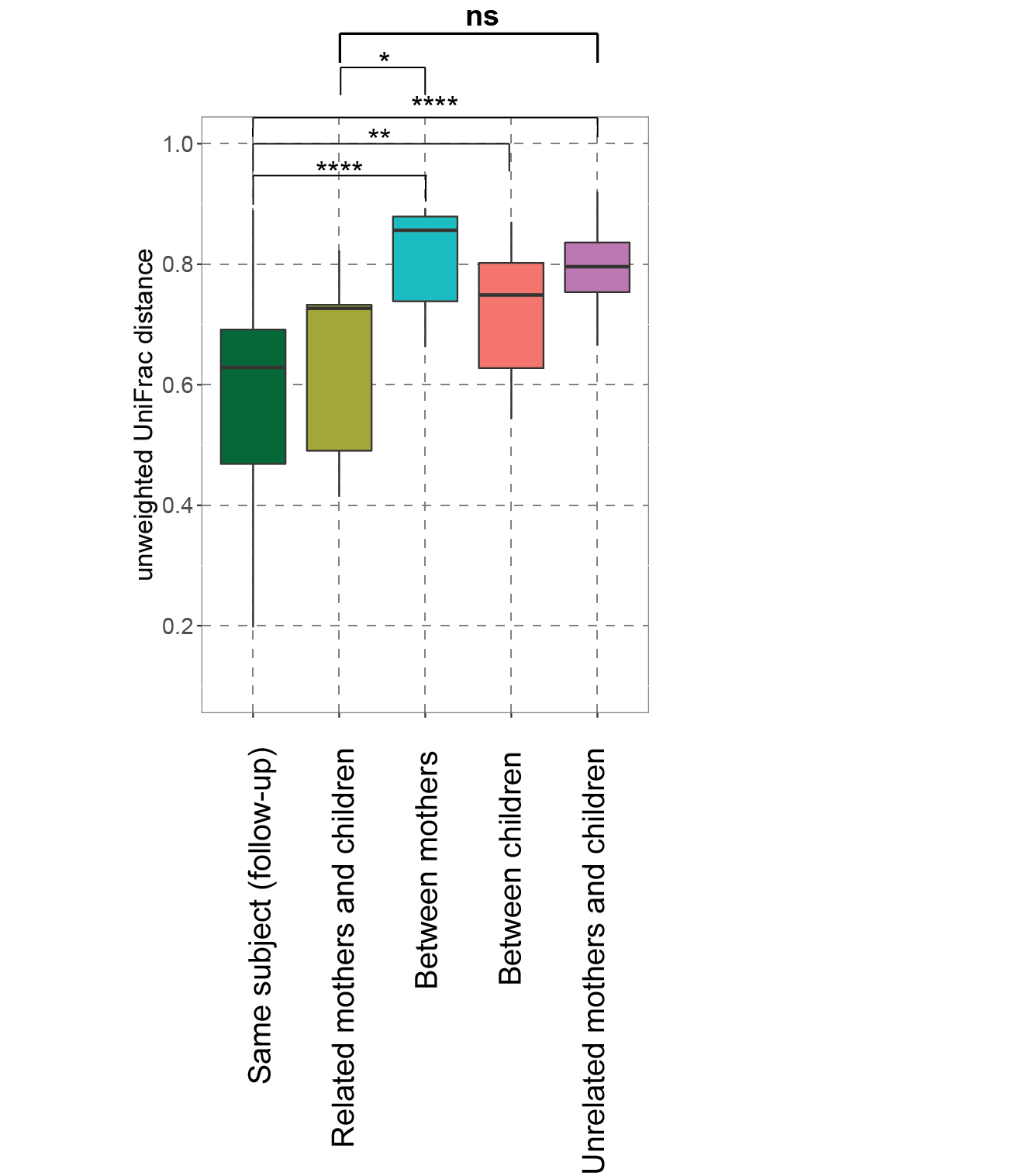


Supplementary Figure S2. Closest to delivery moment mother samples unweighted UniFrac pairwise comparison. comparing related and unrelated samples. The mother samples closest to the delivery moment that were used are indicated in Supplementary Table S1 –marked with a “$” symbol. Statistical significance was assessed using Wilcoxon sum-rank test. Explanations of the symbols: * P ≤ 0.05, ** P ≤ 0.01, *** P ≤ 0.001, **** P ≤ 0.0001, ns – not significant.
